# Advances in the Diagnosis, Treatment and Prognosis of ANCA-Associated Glomerulonephritis

**DOI:** 10.3390/medicina62071252

**Published:** 2026-06-29

**Authors:** Aglaia Chalkia, Dimitrios Petras

**Affiliations:** Nephrology Department, General Hospital of Athens Hippokration, 11527 Athens, Greece

**Keywords:** ANCA-associated vasculitis, ESKD, kidney, prognosis

## Abstract

ANCA-associated vasculitis (AAV) with kidney involvement represents small-vessel vasculitis, characterized by rapidly progressive glomerulonephritis and a high risk of end-stage kidney disease (ESKD) and increased mortality. AAV typically presents with multisystem involvement, with renal manifestations occurring more frequently in microscopic polyangiitis (MPA) (90–100%) and granulomatosis with polyangiitis (GPA) (50–80%). The classic clinical presentation includes acute kidney injury with hematuria and proteinuria, accompanied by ANCA positivity (MPO-ANCA or PR3-ANCA). Histologically, the predominant pattern is segmental necrotizing glomerulonephritis with crescent formation. Treatment consists of two phases: (a) induction of remission with a lower cumulative dose of glucocorticoids (according to the reduced-dose PEXIVAS regimen) in combination with rituximab or cyclophosphamide and (b) maintenance of remission with rituximab for 2–4 years. The C5a receptor inhibitor avacopan can be used as a steroid-sparing agent in patients with severe kidney involvement or at high risk of corticosteroid-related complications. Beyond the traditional markers of disease activity (hematuria, proteinuria, eGFR), novel biomarkers such as urinary soluble CD163, MCP-1, complement activation products (C5a, sC5b-9), and urinary Treg/Th17 profiles have demonstrated prognostic value. Early diagnosis and prompt initiation of immunosuppressive therapy significantly improve both kidney and overall survival, while prevention of relapses and long-term complications plays a key role in improving the long-term prognosis of patients with AAV.

## 1. Introduction

ANCA-associated vasculitides (AAV) are necrotizing small-vessel vasculitides classified into three major clinical phenotypes: microscopic polyangiitis (MPA), granulomatosis with polyangiitis (GPA), and eosinophilic granulomatosis with polyangiitis (EGPA). These disorders are strongly associated with anti-neutrophil cytoplasmic antibodies (ANCA), most commonly directed against myeloperoxidase (MPO) or proteinase-3 (PR3). Kidney involvement represents one of the most severe manifestations of AAV and is a major determinant of both short-term and long-term outcomes. Renal manifestations occur in approximately 90–100% of patients with MPA and in 50–80% of those with GPA [1]. In contrast, kidney disease is less common in EGPA. Although AAV frequently presents with multisystem involvement, approximately 30% of patients have renal-limited disease without overt extrarenal manifestations.

Historically, untreated AAV carried an extremely poor prognosis, with mortality rates approaching 80% within the first year after diagnosis. The introduction of cyclophosphamide and glucocorticoids dramatically improved survival. More recently, biologic therapies such as rituximab and targeted complement inhibition with avacopan have further transformed management strategies [2,3]. Nevertheless, despite major therapeutic advances, many patients continue to develop irreversible kidney injury, and approximately 20–40% progress to end-stage kidney disease (ESKD) within five years of diagnosis [4,5,6].

Histopathologically, kidney involvement in AAV is characterized by pauci-immune necrotizing crescentic glomerulonephritis. Both active inflammatory lesions and chronic irreversible damage may coexist, contributing to the heterogeneity of renal outcomes. In addition to kidney failure, relapses, cardiovascular disease, infections, and treatment-related toxicities remain major causes of morbidity and mortality.

**Figure 1:**
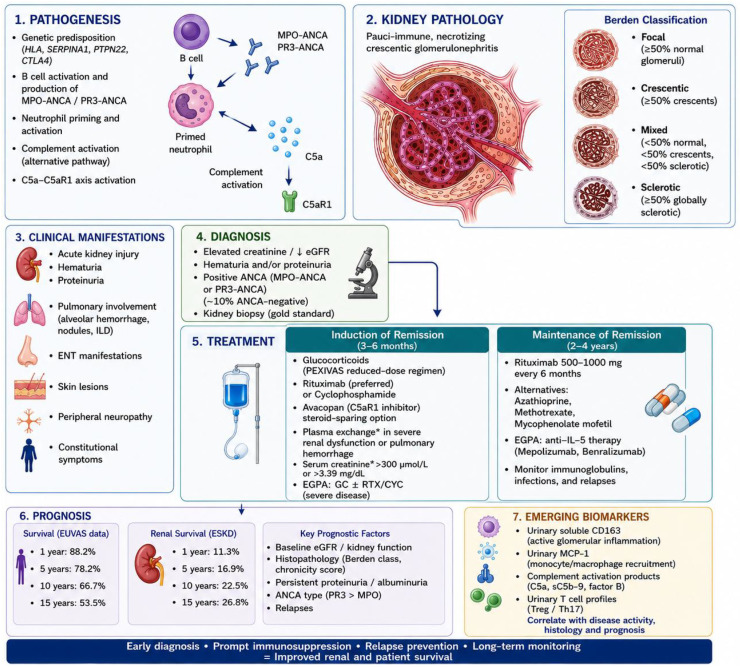
Overview of ANCA-associated Vasculitis with Kidney Involvement. From Pathogenesis to Diagnosis, Treatment and Prognosis. AAV, ANCA-associated vasculitis; ANCA, anti-neutrophil cytoplasmic antibody; MPO, myeloperoxidase; PR3, proteinase 3; eGFR, estimated glomerular filtration rate; ESKD, end-stage kidney disease; ENT, ear–nose–throat; ILD, interstitial lung disease; C5aR, complement 5a receptor; EGPA, Eosinophilic Granulomatosis with Polyangiitis; IL-5, Interleukin 5; MCP-1, monocyte chemoattractant protein-1; Treg, regulatory T cell; Th17, T helper 17 cell. *, indication.

This review focuses specifically on ANCA-associated glomerulonephritis (AAGN), integrating recent advances in diagnosis, novel therapeutic strategies, including avacopan and IL-5-targeted therapies, prognostic risk scores, persistent urinary abnormalities, and emerging biomarkers, with particular emphasis on their potential integration into routine clinical practice (Figure 1).

## 2. Pathogenesis

The pathogenesis of AAV involves a complex interaction between genetic susceptibility, environmental triggers, immune dysregulation, neutrophil activation, and complement pathway activation [7]. ANCAs play a central pathogenic role by activating primed neutrophils and monocytes, leading to endothelial injury and vascular inflammation. Under inflammatory conditions, neutrophils express MPO and PR3 on their cell surface. ANCAs bind to these antigens and induce neutrophil activation, degranulation, production of reactive oxygen species, and release of neutrophil extracellular traps (NETs). Activated neutrophils adhere to vascular endothelium and promote endothelial damage, resulting in fibrinoid necrosis of small vessels. The alternative complement pathway has emerged as another key mechanism in AAV pathogenesis. It can be activated on injured or poorly protected biological surfaces, including activated neutrophils, NETs, apoptotic cells, and damaged endothelium, where regulatory control is reduced. Generated C5a acts as a potent neutrophil chemoattractant and amplifier of inflammation through interaction with the C5a receptor (C5aR). Experimental studies demonstrated that blockade of C5aR attenuates disease activity, providing the rationale for the development of avacopan [8]. Macrophages, T lymphocytes, and B cells also contribute significantly to disease progression. Regulatory T-cell dysfunction and expansion of pro-inflammatory Th17 cells have been implicated in persistent inflammation and relapse susceptibility. MPO-ANCA and PR3-ANCA disease have partly distinct genetic and immunological associations [7]. PR3-ANCA disease is more strongly associated with GPA, granulomatous inflammation, ENT and pulmonary nodular disease, and relapse risk, whereas MPO-ANCA disease is more commonly associated with MPA, kidney involvement, interstitial lung disease, and less frequent relapse.

EGPA has additional pathogenic features, including eosinophilic inflammation, asthma, allergic disease, and tissue eosinophil infiltration. Interleukin-5-driven eosinophil activation contributes to airway inflammation, cardiac and neurologic involvement, and systemic vasculitic manifestations. Thus, EGPA differs from GPA and MPA by combining ANCA-mediated small-vessel vasculitis with eosinophil-dominant inflammation; kidney involvement is less frequent but can occur, particularly in ANCA-positive EGPA [9].

## 3. Clinical Manifestations

### 3.1. Renal Manifestations

Kidney involvement in AAV most commonly presents as rapidly progressive glomerulonephritis (RPGN). Patients typically develop acute kidney injury accompanied by microscopic hematuria, dysmorphic erythrocytes, red blood cell casts, and proteinuria. Proteinuria is usually subnephrotic, although nephrotic-range proteinuria may occasionally occur. The clinical course may vary substantially. Some patients experience rapidly progressive renal dysfunction over days to weeks, while others, particularly those with MPO-ANCA positivity, may present with slowly progressive chronic kidney disease over several months [10]. Severe cases may progress to dialysis-dependent kidney failure at presentation. Persistent microscopic hematuria and proteinuria may remain even after induction therapy [11,12]. Although residual urinary abnormalities may reflect chronic glomerular damage, persistent or increasing proteinuria can also indicate ongoing inflammatory activity or future relapse risk.

Double MPO-ANCA and PR3-ANCA positivity is uncommon and should prompt careful reassessment for assay interference, drug-induced AAV, infection, or overlap syndromes [13]. In patients with pulmonary–renal syndrome, severe anemia, or linear IgG staining on biopsy, anti-glomerular basement membrane (anti-GBM) antibodies should also be tested because double-positive ANCA/anti-GBM disease has a more aggressive early kidney course and requires urgent plasma exchange [14].

### 3.2. Extrarenal Manifestations

AAV commonly affects multiple organ systems simultaneously. Constitutional symptoms such as fever, weight loss, fatigue, and malaise are frequent. Pulmonary involvement is particularly common and may manifest as diffuse alveolar hemorrhage, pulmonary nodules, ground-glass infiltrates, or interstitial lung disease [15]. Diffuse alveolar hemorrhage results from pulmonary capillaritis with necrotizing injury of the alveolar microvasculature, leading to leakage of erythrocytes into alveolar spaces; it may present with hemoptysis, dyspnea, hypoxemia, anemia, and new alveolar infiltrates, although hemoptysis may be absent. Pulmonary involvement in MPA, usually associated with MPO-ANCA, primarily reflects small-vessel capillaritis and presents as DAH (25–60%) or interstitial lung disease (ILD) (20%) [16]. In contrast, pulmonary nodules, masses, or cavitations on CT are more common for GPA-PR3-ANCA-positive [17]. ENT manifestations are especially characteristic of GPA and include chronic sinusitis, nasal crusting, otitis media, subglottic stenosis, and saddle-nose deformity [18]. Cutaneous purpura, peripheral neuropathy, ocular inflammation, arthralgias, and myalgias are also frequently observed. The coexistence of pulmonary hemorrhage and glomerulonephritis represents a life-threatening pulmonary–renal syndrome requiring urgent recognition and aggressive immunosuppressive therapy.

EGPA should be considered in patients with adult-onset asthma, chronic rhinosinusitis, nasal polyposis, peripheral eosinophilia, pulmonary infiltrates, mononeuritis multiplex, skin purpura, or cardiac involvement. ANCA-positive EGPA is more often associated with vasculitic features, including glomerulonephritis and neuropathy, whereas ANCA-negative EGPA is more often associated with eosinophilic tissue disease, particularly cardiac and pulmonary involvement [19].

## 4. Diagnosis

### 4.1. Laboratory Evaluation

The diagnosis of kidney involvement in AAV relies on clinical presentation, laboratory findings, ANCA serology, and kidney biopsy. Laboratory evaluation typically reveals elevated serum creatinine and reduced estimated glomerular filtration rate (eGFR), reflecting impaired kidney function. Urinalysis usually demonstrates microscopic hematuria, dysmorphic red blood cells, red blood cell casts, and proteinuria. Inflammatory markers such as C-reactive protein (CRP) and erythrocyte sedimentation rate (ESR) are frequently elevated. ANCA testing plays a central role in the diagnostic workup. Antigen-specific immunoassays targeting MPO and PR3 have superior diagnostic accuracy compared with indirect immunofluorescence. MPO-ANCA is more commonly associated with MPA, whereas PR3-ANCA is frequently linked with GPA. However, ANCA positivity alone is insufficient for diagnosis, as approximately 10% of patients may be ANCA-negative. Consequently, a negative ANCA test does not exclude AAV. NET formation is biologically important in AAV pathogenesis, but NET-derived markers are not yet standardized or validated for routine diagnostic monitoring [20]; therefore, clinical assessment, urinalysis, kidney function, ANCA specificity, and biopsy remain the main diagnostic tools.

ANCA-negative AAV represents a diagnostic challenge and should be suspected when clinical features and biopsy findings are typical despite negative MPO-ANCA and PR3-ANCA testing [21]. Therefore, kidney biopsy is particularly important to confirm pauci-immune necrotizing crescentic glomerulonephritis and to exclude mimics such as infection-related glomerulonephritis, immune-complex disease, anti-GBM disease, malignancy-associated vasculitis, and drug-induced disease.

### 4.2. Kidney Biopsy

Kidney biopsy remains the gold standard for confirming ANCA-associated glomerulonephritis and provides critical prognostic information. The hallmark lesion is pauci-immune necrotizing crescentic glomerulonephritis characterized by fibrinoid necrosis, segmental glomerular destruction, and crescent formation with minimal immune complex deposition. The Berden classification categorizes ANCA-associated glomerulonephritis into four histopathological classes [22]. The focal class is defined by the presence of at least 50% normal glomeruli and is associated with the best renal prognosis. The crescentic class is characterized by at least 50% glomeruli containing cellular crescents and generally reflects active inflammatory disease with potential reversibility. The mixed class demonstrates a heterogeneous pattern of lesions, i.e., <50% normal, <50% crescentic, and <50% globally sclerotic glomeruli, whereas the sclerotic class, defined by at least 50% globally sclerotic glomeruli, is associated with severe chronic damage and poor renal recovery.

## 5. Treatment

### 5.1. MPA/GPA

#### 5.1.1. Induction Treatment

Induction therapy for severe GPA and MPA, including glomerulonephritis, is based on glucocorticoids combined with either rituximab or cyclophosphamide [3,23,24,25,26] (Table 1). Rituximab is generally preferred in relapsing disease, supported by the RAVE and RITAZAREM trials [24,27]. Combination therapy with rituximab and low-dose cyclophosphamide demonstrated efficacy comparable to standard-dose cyclophosphamide in the RITUXVAS study [23] and can be used in patients with severe kidney impairment (serum creatinine > 4 mg/dL), as cyclophosphamide may exert a more rapid effect than rituximab [3].

Although the PEXIVAS trial [28], which included patients with severe kidney involvement (eGFR < 50 mL/min/1.73 m^2^), did not demonstrate a reduction in the composite outcome of ESKD or death with plasma exchange, a subsequent meta-analysis [29] suggested that plasma exchange may reduce the short-term risk of progression to ESKD in selected high-risk patients. Consequently, both the EULAR recommendations and KDIGO guidelines suggest that plasma exchange may be considered in patients with severe kidney dysfunction (serum creatinine > 300 μmol/L or >3.39 mg/dL). However, this potential benefit should be balanced against the increased risk of serious infections, emphasizing the importance of careful patient selection. A potential survival benefit has also been suggested in patients with severe pulmonary hemorrhage and hypoxemia in a subgroup analysis of the PEXIVAS trial [30].

The PEXIVAS and LOVAS studies [28,31] substantially modified glucocorticoid treatment strategies. Reduced-dose glucocorticoid regimens demonstrated similar efficacy to standard regimens with fewer severe infections. The ADVOCATE trial [25] demonstrated that avacopan, a selective C5a receptor inhibitor, achieved higher rates of sustained remission at 12 months, improved quality of life, higher recovery of kidney function and fewer glucocorticoid-related toxicities compared with standard glucocorticoid therapy. Among patients with kidney involvement, avacopan was associated with faster reductions in albuminuria and greater improvements in eGFR, particularly in those with advanced kidney dysfunction [32]. Real-world evidence supports the efficacy and favorable safety profile of avacopan in patients with AAV and kidney involvement, with high remission rates achieved despite rapid glucocorticoid discontinuation [33,34]. Additionally, avacopan has shown promising results in critically ill patients, including those with hypoxic pulmonary hemorrhage [35,36] and patients with eGFR < 15 mL/min/1.73 m^2^ [33].

#### 5.1.2. Maintenance Treatment

Maintenance therapy has progressively shifted from azathioprine or methotrexate toward rituximab, supported by the MAINRITSAN and RITAZAREM studies [27,37] (Table 1). Rituximab administered every six months for 2–4 years is currently recommended, although some patients may require intensified regimens. Long-term rituximab therapy effectively reduces relapse risk but is associated with complications such as infections and hypogammaglobulinemia, necessitating regular immunoglobulin monitoring.

### 5.2. EGPA

In EGPA, treatment is guided by disease severity. Patients with severe or organ-threatening manifestations, including glomerulonephritis, are treated with glucocorticoids combined with cyclophosphamide or rituximab, whereas glucocorticoid monotherapy may be sufficient in non-severe disease. The REOVAS trial showed that rituximab was not superior to conventional treatment strategies for remission induction [38]. For maintenance therapy, rituximab is frequently used, although evidence is mainly derived from observational studies. More recently, IL-5-targeted therapies have expanded treatment options. Mepolizumab and benralizumab have demonstrated efficacy in relapsing or refractory EGPA, reducing relapse rates and glucocorticoid exposure. In the MIRRA trial, mepolizumab prolonged remission and reduced relapses, while the MANDARA trial demonstrated that benralizumab was non-inferior to mepolizumab for achieving remission [39,40].

## 6. Prognosis

### 6.1. Survival

Early recognition and prompt initiation of immunosuppressive therapy have dramatically improved outcomes in AAV. Before the introduction of immunosuppressive treatment, one-year mortality approached 80% [41]. The introduction of cyclophosphamide-based regimens and, more recently, rituximab and targeted therapies has significantly improved both patient and renal survival. Currently, five-year survival exceeds 80–90%, although substantial morbidity and chronic complications remain common. Data from the EUVAS cohorts demonstrated overall survival rates of 88.2%, 78.2%, 66.7%, and 53.5% at 1, 5, 10, and 15 years, respectively [42]. Despite these therapeutic advances, mortality in AAV remains higher than in the general population, particularly among patients with severe renal involvement, advanced age, or significant comorbidities. Several clinical and laboratory parameters have been identified as important prognostic factors. Advanced age, male sex, low estimated glomerular filtration rate (eGFR), and low platelet count at diagnosis are associated with poorer overall survival. In addition, severe pulmonary involvement, diffuse alveolar hemorrhage, and the need for dialysis at presentation are linked with increased early mortality. The major causes of death have evolved over time. During the early phase of disease, mortality is predominantly related to active vasculitis and severe infections resulting from intensive immunosuppressive therapy. In contrast, cardiovascular disease, chronic kidney disease, malignancies, and infectious complications become increasingly important during long-term follow-up. Chronic systemic inflammation, endothelial dysfunction, glucocorticoid exposure, and persistent renal impairment contribute substantially to accelerated cardiovascular risk in AAV patients.

### 6.2. Renal Survival

Renal survival remains a major concern, with ESKD rates of 11.3%, 16.9%, 22.5%, and 26.8% at 1, 5, 10, and 15 years, respectively [6]. Several factors have been associated with poor renal prognosis, including age greater than 65 years, severely reduced eGFR at diagnosis, and low hemoglobin levels. Patients presenting with advanced kidney dysfunction or dialysis dependency are at particularly high risk for progression to irreversible kidney failure. Nevertheless, partial renal recovery may still occur in some dialysis-dependent patients if aggressive treatment is initiated early, emphasizing the importance of rapid diagnosis and treatment initiation.

Three clinicopathological risk scores have been developed to predict progression to ESKD [43]. The Berden classification focuses on glomerular lesions, whereas the Mayo Clinic Chronicity Score incorporates chronic changes across all renal compartments [44]. The Improved Kidney Risk Score in ANCA-Associated Vasculitis (AKRiS) integrates baseline kidney function with histopathological findings to stratify ESKD risk [45]. Although these tools provide prognostic information, they are not yet routinely used to guide therapeutic decisions. Glomerular hematuria and proteinuria are early markers of glomerular injury and frequently precede declines in eGFR. However, persistent hematuria and/or proteinuria are common following induction therapy in AAGN. Persistent proteinuria or albuminuria has been consistently associated with an increased risk of progression to ESKD [11,12], while persistent hematuria has been linked to an elevated risk of renal relapse [12]. However, interpretation of persistent urinary abnormalities remains challenging in clinical practice, as both proteinuria and hematuria may reflect ongoing inflammatory activity or chronic irreversible damage. Therefore, these markers should be interpreted together with trends in kidney function and, when available, histopathological findings.

## 7. Relapse

Relapses are frequent in AAV and significantly influence long-term organ function and cumulative tissue damage. Patients with PR3-ANCA positivity, pulmonary involvement, or ENT manifestations exhibit higher relapse rates compared with those with MPO-ANCA positivity or renal-limited disease [46,47]. Relapses may involve the kidneys or extra-renal organs and often require escalation or reintroduction of immunosuppressive therapy. Rituximab substantially reduces relapse risk, although relapses may still occur and occasionally require escalation of immunosuppressive therapy [48]. Long-term rituximab therapy is highly effective but may be associated with hypogammaglobulinemia and increased susceptibility to infections, particularly in elderly patients or those with chronic kidney disease. Even among patients receiving chronic dialysis, the risk of extrarenal relapse persists, supporting continuation of maintenance immunosuppression in selected cases [49]. However, the benefits of prolonged immunosuppression must be carefully balanced against the increased risks of severe infections, malignancies, and cardiovascular complications [50].

## 8. Emerging Biomarkers

Recent advances in understanding AAV pathogenesis have led to the identification of novel biomarkers with potential prognostic value (Table 2). Urinary soluble CD163 has emerged as one of the most promising biomarkers of active AAGN [51,52,53]. CD163 is a scavenger receptor shed by activated M2 macrophages, and elevated urinary levels strongly correlate with active glomerular lesions, particularly crescentic disease. Levels decrease significantly during remission and appear to outperform conventional biomarkers in detecting renal relapse. Urinary monocyte chemoattractant protein-1 (MCP-1), a key chemokine involved in monocyte and macrophage recruitment, is also elevated during active disease and progressively decreases with treatment [25]. Complement activation products, including plasma C3a, C5a, factor B, and urinary soluble C5b-9, have also been associated with disease activity and histological severity [54]. Finally, urinary T-cell profiles, particularly regulatory T cells (Treg) and Th17 cells, have demonstrated significant correlations with clinical activity and may provide superior predictive value compared with traditional urinary biomarkers [55].

Urinary biomarkers are typically normalized to urinary creatinine and interpreted alongside eGFR, proteinuria, hematuria, treatment status, and histopathological findings. They may help identify active renal inflammation, assess relapse risk, and monitor treatment response. However, most remain investigational, as assays are not standardized and prospective validation is still required before routine clinical use.

## 9. Future Perspectives

Future management strategies in AAV are expected to increasingly focus on personalized medicine approaches integrating clinical, histopathological, serological, and molecular data. Several novel therapeutic approaches are currently under investigation and may further improve outcomes while reducing treatment-related toxicity. These include newer complement-directed therapies (e.g., factor B inhibition), next-generation B-cell-depleting agents (e.g., obinutuzumab), plasma cell-targeted therapies (e.g., daratumumab and CAR-T cell therapy), agents targeting B-cell cytokines (e.g., BLys and APRIL inhibitors), and therapies modulating T-cell activation (e.g., abatacept and ustekinumab) [56,57]. Biomarker-guided treatment strategies could allow more individualized decisions regarding induction treatment, maintenance duration, and relapse prevention. Advances in artificial intelligence and machine learning may also improve prognostic modeling and facilitate earlier identification of patients at high risk for ESKD or recurrent disease activity [58]. Continued research into the mechanisms of immune dysregulation and chronic kidney injury remains essential for the development of safer and more effective therapeutic strategies.

## 10. Conclusions

ANCA-associated vasculitis with kidney involvement remains a severe and potentially life-threatening condition associated with substantial risks of ESKD and mortality. Early diagnosis and prompt initiation of immunosuppressive therapy are critical for preventing irreversible kidney damage and improving patient survival. Recognition of characteristic clinical features, including rapidly progressive kidney dysfunction, hematuria, proteinuria, and multisystem involvement, together with histopathological findings of necrotizing crescentic glomerulonephritis, enables timely diagnosis and treatment initiation. The introduction of targeted therapies such as the C5a receptor inhibitor avacopan offers the opportunity to reduce glucocorticoid exposure and related toxicities, particularly in patients with severe kidney disease or increased risk of steroid-related complications. At the same time, established induction and maintenance regimens based on rituximab and cyclophosphamide remain fundamental for achieving durable remission and reducing relapse risk. Emerging biomarkers, including urinary soluble CD163, MCP-1, complement activation products, and urinary Treg/Th17 profiles, may enhance disease monitoring and prognostic assessment. Overall, the integration of clinical, histopathological, and molecular markers with both targeted and conventional therapeutic approaches is expected to further improve renal and patient survival in AAV.

## Figures and Tables

**Table 1:** Current Therapeutic Strategies in ANCA-Associated Vasculitis with Kidney Involvement.

Therapy	Indications	Mechanism of Action	Major Benefits	Main Limitations/Adverse Effects	Key Supporting Trials
Glucocorticoids	Induction therapy in active disease	Broad anti-inflammatory and immunosuppressive effects	Rapid disease control and symptom improvement	Infections, diabetes, osteoporosis, cardiovascular toxicity	PEXIVAS, LOVAS
Rituximab	Induction and maintenance therapy, particularly relapsing disease	Anti-CD20 B-cell depletion	Effective relapse prevention and steroid-sparing potential	Hypogammaglobulinemia and infections	RAVE, RITUXVAS, RITAZAREM, MAINRITSAN
Cyclophosphamide	Induction therapy Severe kidney disease or life-threatening vasculitis	Cytotoxic suppression of immune cells	Potent induction efficacy in severe disease	Cytopenias, infertility, malignancy risk	CYCLOPS, RITUXVAS
Plasma Exchange	Severe kidney dysfunction or diffuse alveolar hemorrhage	Removal of circulating inflammatory mediators and ANCAs	Possible reduction in ESKD progression	Increased infection risk and uncertain long-term benefit	MEPEX PEXIVAS
Avacopan	Severe kidney involvement or glucocorticoid toxicity risk	Selective C5a receptor inhibition	Reduced glucocorticoid exposure and improved renal recovery	High cost and limited long-term experience	ADVOCATE
Azathioprine	Alternative maintenance therapy	Purine synthesis inhibition	Oral administration and long clinical experience	Less effective relapse prevention compared with rituximab	CYCAZAREM

ESKD; end-stage kidney disease, ANCA, anti-neutrophil cytoplasmic antibody; C5a, complement component 5a; CD20, cluster of differentiation 20.

**Table 2:** Emerging Biomarkers and Prognostic Factors in ANCA-Associated Vasculitis with Kidney Involvement.

Biomarker/Factor	Biological Significance	Clinical Association	Potential Clinical Utility
Urinary soluble CD163	Marker of activated macrophages	Correlates with active crescentic glomerulonephritis	Detection of active renal vasculitis and relapse monitoring
MCP-1	Monocyte and macrophage recruitment chemokine	Elevated during active renal inflammation	Assessment of treatment response
Plasma C5a	Alternative complement pathway activation	Associated with disease activity and severity	Identification of patients who may benefit from complement inhibition
Urinary soluble C5b-9	Terminal complement activation marker	Correlates with histological severity	Prognostic biomarker for renal injury
Persistent proteinuria	Reflects chronic glomerular injury	Associated with progression to ESKD	Long-term renal prognosis
Persistent hematuria	Indicates ongoing glomerular inflammation	Linked with increased renal relapse risk	Disease activity monitoring
PR3-ANCA positivity	Distinct immunologic phenotype	Higher relapse frequency	Risk stratification and maintenance therapy decisions
Reduced baseline eGFR	Severe kidney dysfunction at presentation	Poor renal survival	Prognostic stratification
Treg/Th17 imbalance	Immune dysregulation	Correlates with active disease	Potential future personalized monitoring tool

CD163; cluster of differentiation 163, MCP-1, monocyte chemoattractant protein-1; C5a, complement component 5a; C5b-9, soluble membrane attack complex; PR3, proteinase 3; eGFR, estimated glomerular filtration rate; ESKD, end-stage kidney disease; Treg, regulatory T cell; Th17, T helper 17 cell; ANCA, anti-neutrophil cytoplasmic antibody.

## Data Availability

No new data were generated or analyzed in support of this research.
